# Genetic landscape of Italian SRLV: insights from passive surveillance-based phylogenetic analysis

**DOI:** 10.3389/fmicb.2025.1745687

**Published:** 2026-01-09

**Authors:** Paola Gobbi, Luca Tassoni, Elena Tinelli, Eleonora Scoccia, Sara Mrabet, Mario Orrico, Cecilia Righi, Carmen Maresca, Nicoletta D’Avino, Fabrizio Passamonti, Monica Giammarioli, Francesco Feliziani, Maria Serena Beato

**Affiliations:** 1 National Reference Laboratory for Ruminant Retroviruses (CEREL), Istituto Zooprofilattico Sperimentale dell’Umbria e delle Marche “Togo Rosati” (IZSUM), Perugia, Italy; 2 Department of Veterinary Medicine, University of Perugia, Perugia, Italy; 3 Department of Epidemiology, Istituto Zooprofilattico Sperimentale dell’Umbria e delle Marche “Togo Rosati” (IZSUM), Perugia, Italy; 4 Istituto Zooprofilattico Sperimentale del Piemonte, Liguria e Valle d’Aosta, Torino, Italy; 5 Small Ruminant Pathology Centre, Istituto Zooprofilattico Sperimentale dell’Umbria e delle Marche “Togo Rosati” (IZSUM), Perugia, Italy

**Keywords:** genetic classification, genetic variability, molecular characterization, phylogenetic analysis, SRLV

## Abstract

Small ruminant lentiviruses (SRLVs) are retroviruses infecting small ruminants, characterized by a high genetic variability. SRLVs infection may result in a variety of clinical signs in goats and sheep and is not species-specific. SRLVs are worldwide distributed with four distinct genotypes and numerous sub-genotypes described so far. Full understanding of SRLVs epidemiology is still a matter of research and genetic characterization remains a key tool for unrevealing the complexity of such infections. A previous study, exploring the genetic landscape of SRLV in Italy, reported considerable genetic heterogeneity across geographic regions and suggested the presence of new sub-genotypes. In light of these previous results, our study provides an update on the genetic characterization of SRLVs based on phylogenetic analysis of SRLVs positive samples collected during passive surveillance in 16 out of 20 Italian regions between 2019 and 2024. Our findings revealed for the first time the broad genetic diversity of SRLVs circulating across Italy with apparently restricted circulation of some sub-genotypes to specific areas/regions. The observed genetic clusters are region-specific and do not align with the currently recognized sub-genotype nomenclature, suggesting that the existing classification system may necessitate an update to describe the current SRLVs diversity more accurately. Continuous monitoring through full gene and genome sequencing, coupled with an updated and expanded genetic classification framework, is essential for improving our understanding of this complex and evolving viral infection in small ruminants.

## 1 Introduction

Small ruminant lentiviruses (SRLVs) are small-enveloped RNA viruses (80–100 nm) that belong to the *Retroviridae* family, subfamily *Orthoretrovirinae*, genus Lentivirus. Initially, SRLVs were described as Maedi-Visna virus (MVV) and caprine arthritis-encephalitis virus (CAEV), in sheep and goats, respectively (Cowdry and Marsh, 1927; Sigurdardóttir Bergthóra, 1964). Animals infected with MVV primarily display respiratory and nervous clinical signs, while animals infected with CAEV primarily exhibit joint damages and nervous clinical signs (Gomez-Lucia et al., 2018). The infection may remain subclinical for a long time (Nalbert et al., 2020; Peterhans et al., 2004), with proviral DNA persisting in monocytes, evading the host immune system before disseminating to target organs such as lung, joints, nervous system and mammary gland. Several clinical forms are described, such as pulmonary, articular, nervous and mammary (Peterhans et al., 2004) with severity depending on viral factors and immune status of infected animals (Pinczowski et al., 2017). The main route of transmission is horizontal, through aerosol and direct contact (Blacklaws et al., 2004; Kalogianni et al., 2020). Vertical routes such as germ line, intrauterine/transplacental infection or lactogenic transmission may occur (Pisoni et al., 2010). Retroviruses are characterized by the ability to reversely transcribe the viral RNA to double stranded DNA (dsDNA) through the action of reverse transcriptase (RT). The virion structure comprises nucleocapsid (NC), capsid (CA), matrix (MA) and envelope (ENV). The genome contains two identical molecules of ssRNA+, included in the NC. The virus envelope is formed by a lipid bilayer of cellular origin and the ENV glycoproteins: transmembrane (TM), in a transmembrane position, and surface (SU), projecting outwards (Minguijón et al., 2015). The SRLV genome organization, common to all lentiviruses, with the genome of the provirus presenting a non-coding long terminal repeat (LTR) at both ends, and between the two LTRs there are three structural genes: *gag*, encoding NC, CA and MA proteins, *pol*, encoding RT, PR and IN, and *env*, encoding the TM and SU glycoproteins. In addition, SRLVs also encode accessory genes: *vif*, *vpr-like* and *rev* (Villet et al., 2003; Gifford, 2012). *Gag* and *pol* genes are widely used as targets for sequencing and phylogenetic analysis (Shah et al., 2004a; Michiels et al., 2020; Molaee et al., 2020). The current SRLVs genetic classification is based on the phylogenetic characterization proposed by Shah et al. (2004a) based on DNA pairwise distance analysis. The classification adopts the nucleotide differences at the *gag-pol* genetic region to differentiate four genotypes (A-B-C-E) and numerous sub-genotypes: 27 for genotype A, five for B, and two for E (Bazzucchi et al., 2021; Olech, 2023), highlighting an exceptional genetic variability and diversity. The previously identified D genotype has been discussed and is now associated with genotype A (Bazzucchi et al., 2021; Ramírez et al., 2013). The A genotype SRLVs are also identified as MVV-like viruses, while the B genotype strains are CAEV-like virus strains. Nowadays, interspecies transmission has been widely demonstrated, and A and B genotypes are detected both in goats and sheep (Shah et al., 2004b; Caroline et al., 2010; Olech and Kuźmak, 2021; Braz et al., 2022). SRLVs are widespread globally and were reported in all five continents with the lowest prevalence in South and central America and the highest prevalence in Europe with all countries affected (de Miguel et al., 2021; World Animal Health Information System, n.d.). Data available on SRLVs distribution and characterization in Italy from 1998 to 2019 indicated the circulation of three genotypes (A, B, and E) and 14 sub-genotypes, all belonging to the A and B genotypes. In particular three B sub-genotypes were described (Bazzucchi et al., 2021; Arcangeli et al., 2022), all co-circulating in central Italy, and 11 A sub-genotypes, with two never described before in Italy, namely A3 and A5 and two new sub-genotypes namely A23 and A24 (Bazzucchi et al., 2021) were suggested, while an Italian SRLV previously described as A9 (Grego et al., 2007) was reclassified as A24 (Bazzucchi et al., 2021; Ostuni et al., 2024). Drawing upon these findings, in this study we conducted a phylogenetic analysis of SRLV in Italy, using data obtained through 5 years of passive surveillance activities across 16 out of 20 Italian regions. The objective was to identify circulating genetic groups and to assess the presence of known and/or novel sub-genotypes. Our results will contribute to improving the understanding of the virus molecular epidemiology and may support the development of more targeted control strategies.

## 2 Materials and methods

### 2.1 Sample collection

Samples included in the study were collected from passive surveillance conducted between October 2019 and October 2024 in Italy. Samples and carcasses from sheep and goats were submitted by private veterinarians directly to the National Reference Laboratory for Ruminant Retroviruses (CEREL) of the Istituto Zooprofilattico Sperimentale dell’Umbria e delle Marche (IZSUM) or to peripheral IZSUM diagnostic laboratories, or by other veterinary institutes in Italy, based on clinical suspicions for SRLV detection. A descriptive analysis of collected data such as origin was performed, and the qualitative and quantitative variables are displayed in tables and graphs. The carcasses were conferred to the laboratory for diagnostic confirmation.

### 2.2 Detection and characterization of SRLV from sheep and goat samples

#### 2.2.1 Sample preparation and DNA/RNA extraction

Biological samples were collected from both sheep and goats. For SRLV screening by PCR one or more samples per animal were tested. DNA or RNA extraction was performed, depending on the available biological matrix (DNA from buffy coat, blood clot, udder homogenate and milk, RNA from other tissues/organs homogenate and milk). In the case of tissues/organs, a section (about 5 mm^3^) was homogenized in 1 mL of phosphate-buffered saline (PBS) supplemented with antibiotics (PBS-A: 10,000 IU/mL penicillin G, 10 mg/mL streptomycin), using tissue lyser (QIAGEN, Hilden, Germany) at 20 Hz for 10 min. Somatic cells from milk were obtained by centrifugation at 3,000 × g for 30 min, then fat layer and supernatant were removed. Finally, the somatic cell pellet was re-suspended in PBS-A. DNA extraction was performed manually using the following kits, according to the manufacturer’s instructions: QIAamp DNA Mini Kit (Qiagen, Hilden, Germany) and High Pure PCR Template Preparation Kit (Roche, Mannheim, Germany). Viral RNA extraction was performed manually according to the manufacturer’s instructions, using the QIAamp Viral RNA Mini Kit-Spin Protocol (Qiagen, Hilden, Germany). Alternatively, the automated QIAsymphony SP/AS extractor with the Pathogen Mini kit (Qiagen, Hilden, Germany) was used for both types of matrices, following the Complex 200 OBL protocol. Homogenates and eluted DNA/RNA were stored at −80 °C until use.

#### 2.2.2 SRLV detection by nested PCR

For samples obtained from RNA-extracted matrices, the amplification protocol included an initial reverse transcription phase, followed by a nested PCR targeting the *gag-pol* region, adapted from Grego et al. (2007), while eluted DNA was used directly for the *gag-pol* nested PCR, using 1 μg of DNA per PCR reaction. The *gag-pol* gene encode for viral nucleocapsid proteins and reverse transcriptase, respectively, and they were used as targets for genotypes A and B. A 1,300 bp fragment was amplified in the first round, using the following primer sequences: GAGF1 for 5′-TGGTGARKCTAGMTAGAGACATGG-3′ and POLR1 rev 5′-CATAGGRGGHGCGGACGGCASCA-3′. Templates from the first PCR were tested to amplify an 800 bp fragment in the second round, using the following primer sequences: GAGF2 for 5′-CAAACWGTRGCAATGCAGCATGG-3′ and POLR2 rev 5′-GCGGACGGCASCACACG-3′. The reverse transcriptase mix was carried out in 20 μL reaction volume consisting of 4 μL of Strand Buffer 5X, 1 μL of dNTPs 10 mM (ThermoFisher Scientific, Massachusetts, United States), 1 μL of random primers 50 μM (Promega, Wisconsin, United States), 0.25 μL of RNAse Inhibitor 40 U/μL (Promega, Wisconsin, United States), 0.5 μL of SuperScript^™^ III enzyme 200 U/μL (ThermoFisher Scientific, Massachusetts, United States) and 5 μL of RNA. The reverse transcriptase mix was incubated in a thermocycler at 37 °C for 90 min (min), followed by 65 °C for 10 min. The first round of the nested PCR was carried out in 50 μL reaction volume consisting of 5 μL of Buffer 10X, 2 μL of MgCl2 25 mM, 1 μL of dNTPs 10 mM (ThermoFisher Scientific, Massachusetts, United States), 1.5 μL of each primer (20 μM), 5 μL of BSA 10X, 0.2 of Taq Platinum^™^ DNA Polymerase (Invitrogen Corporation, United States) and 3 μL of cDNA or 10 μL of DNA. The PCR program used had the following thermal profile: Taq polymerase activation at 94 °C for 2 min, followed by 40 cycles with denaturation at 94 °C for 45 s, annealing at 55 °C for 1 min and extension at 72 °C for 1 min, followed by 10 min at 72 °C. The second round of the nested PCR was carried out in 50 μL reaction volume consisting of the same volumes from the first round PCR and 3 μL of template. The PCR program used had the following thermal profile: Taq polymerase activation at 94 °C for 2 min, followed by 40 cycles with denaturation at 94 °C for 45 s, annealing at 65 °C for 1 min and extension at 72 °C for 1 min, followed by 10 min at 72 °C. All the volume adjustments were made with H_2_O + DEPC. The MVV 1514 strain was used as a positive control for genotype A, and CAEV-Co for genotype B. PCR results were analyzed via electrophoresis on a 2% agarose gel, using Midori Green Advance (NIPPON Genetics Europe GmbH, Düren, Germany) as an intercalating agent.

#### 2.2.3 Sanger sequencing of the *gag-pol* gene segment

The *gag-pol* gene of all SRLV positive samples, one per tested animal, detected between 2019 and 2024 were subjected to Sanger sequencing. Following the nested *gag-pol* PCR as described above, bands of the expected size of 800 bp were excised from agarose gel and purified using the QIAquick Gel Extraction kit (Qiagen, Hilden, Germany) following the manufacturer’s protocol. The purification product was used as a template in the sequencing reaction (chain termination PCR) conducted using the BrilliantDye^™^ Terminator Cycle Sequencing v3.1 Kit (NimaGen BV, Nijmegen, The Netherlands). Two replicates of the sequencing reaction were set up for each sample using the forward primer (GAG-F2) and two replicates using the reverse primer (POL-R2). Both GAG-F2 and POL-R2 were used at a concentration of 3.2 μM. The prepared reaction mixes were loaded into the thermocycler and incubated according to the following thermal profile: Taq polymerase activation at 96 °C for 45 s, followed by 25 cycles with denaturation at 96 °C for 10 s, annealing at 50 °C for 5 s and extension at 60 °C for 4 min. The sequencing reaction products were subsequently precipitated using the DyeEx 2.0 Spin Kit (Qiagen, Hilden, Germany) following the manufacturer’s protocol and denatured at 95 °C for 5 min after the addition of HiDi Formamide (Applied Biosystems Life Technologies, Warrington, United Kingdom). Following denaturation, the products were loaded into the ABI PRISM 3130 Genetic Analyzer automatic sequencer (Applied Biosystems, Foster City, CA, United States). The forward and reverse sequences of each sample were analyzed and assembled using DNAStar Navigator software v.15, yielding the consensus sequence, which was then compared and aligned with other reference sequences downloaded from GenBank and utilized for genotyping. The aligned sequences were further refined and trimmed using BioEdit v.7.2.5 (Hall, 1999), Seqscape v3.0 (ThermoFisher, Waltham, Massachusetts, United States), MEGA X (Kumar et al., 1994) and FinchTV v1.4.0 (Geospiza, United States). Sequence data generated in the present work were submitted to Bankit NCBI database, under accession number from OR669734 to OR669737, from OR743469 to OR743492, from PP471985 to PP471993, from PP501837 to PP501870 and from PQ866022 to PQ866040.

### 2.3 Phylogenetic analysis

Phylogenetic analysis was performed on *gag-pol* sequences of positive samples collected from the period 1 October 2019 to 31 October 2024. Phylogenetic analyses were performed separately for genotype A and genotype B sequences. After a preliminary analysis to identify the genotype, the sequences obtained were divided into two datasets based on their genotype (A or B). The datasets were enriched with the addition of other Italian sequences described and classified in the literature as well as with sequences that were found to be similar based on a BLAST search (Bazzucchi et al., 2021; Arcangeli et al., 2022; Altschul et al., 1990). Sequence alignment was carried out using MAFFT and subsequently manually corrected in MEGA X (Katoh et al., 2018; Kumar et al., 2018). Phylogenetic analysis was then carried out separately on the two datasets using the maximum likelihood algorithm. Three different software packages, IQ-TREE 2, RAxML and PhyML 3.0, were used to generate the trees and confirm the identified topologies (Minh et al., 2020; Edler et al., 2021; Guindon et al., 2010). The trees obtained were visualized with FigTree v1.4.3 and the corresponding figures prepared with Inkscape (Inkscape, 2024; Molecular Evolution, Phylogenetics and Epidemiology, 2024). The presence of any recombination events between different subtypes was tested using the RDP5 software (Martin et al., 2021). Consensus sequences generated for this study were uploaded in Genbank (Supplementary Table 1).

## 3 Results

### 3.1 Spatial–temporal distribution of SRLVs in Italy

**Figure 1:**
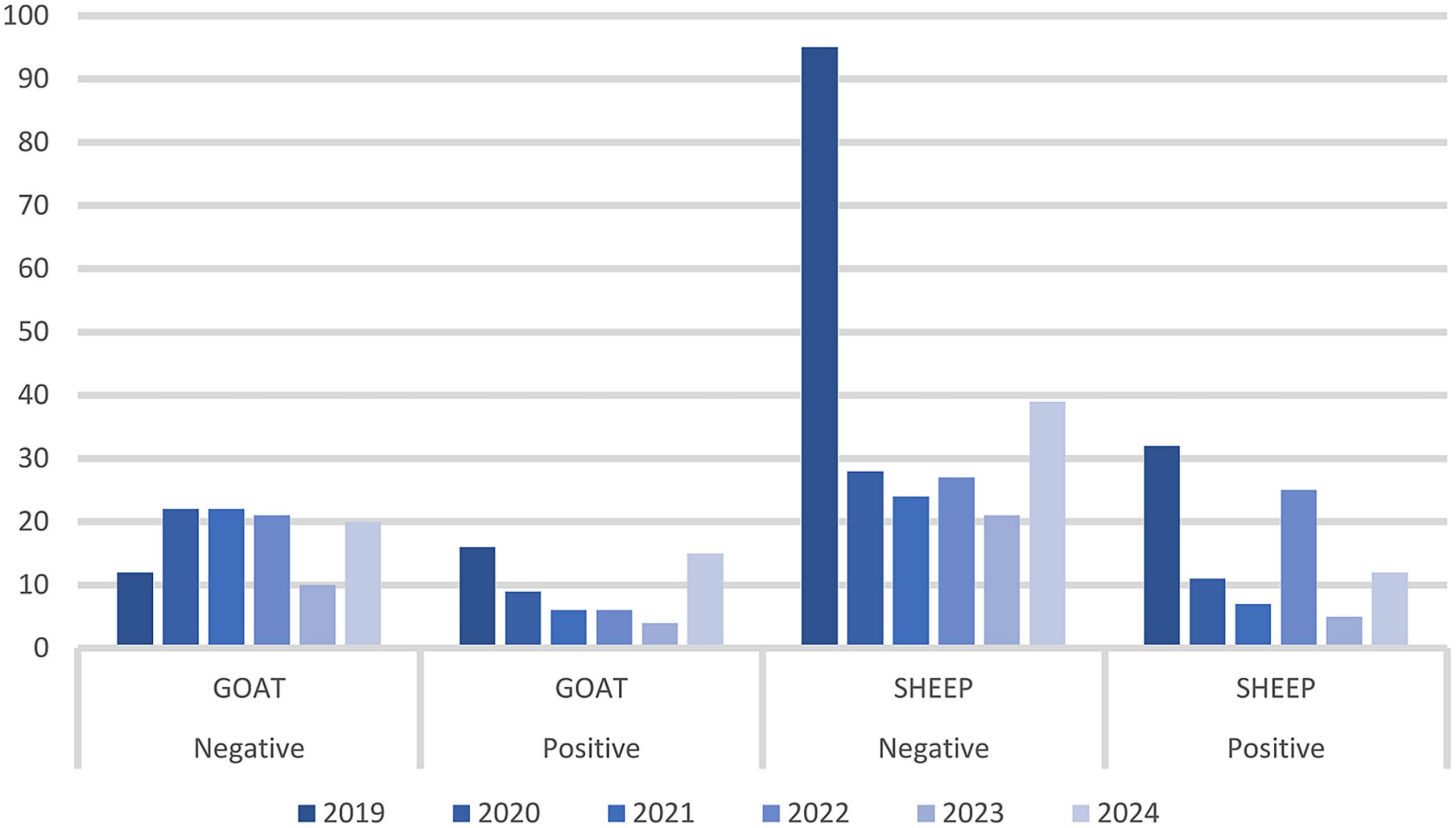
Samples analyzed per year, species, and results. The number of samples tested is reported on the *Y*-axis.

**Figure 2:**
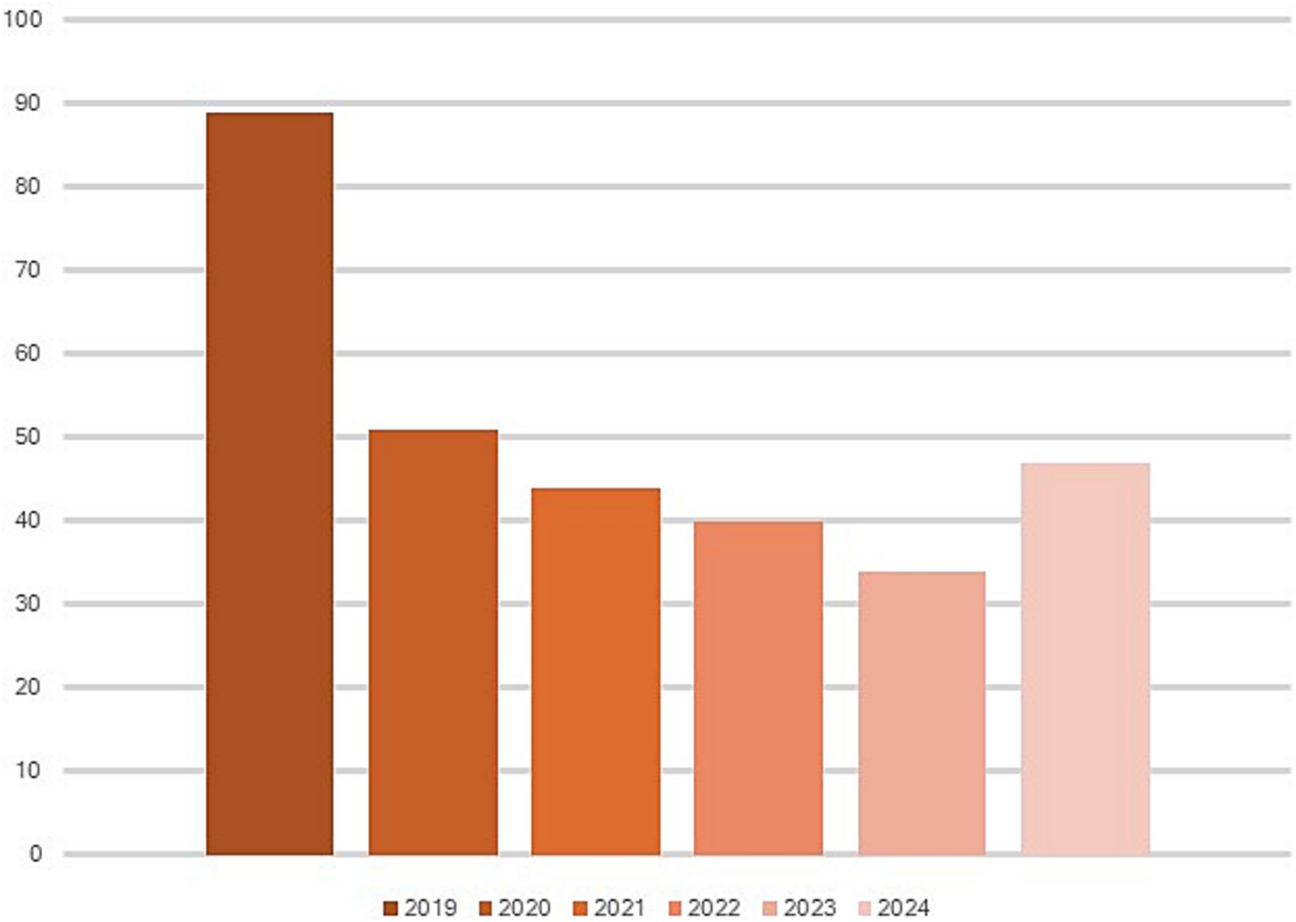
Tested farms per year. The number of tested farms is reported on the *Y*-axis.

**Figure 3:**
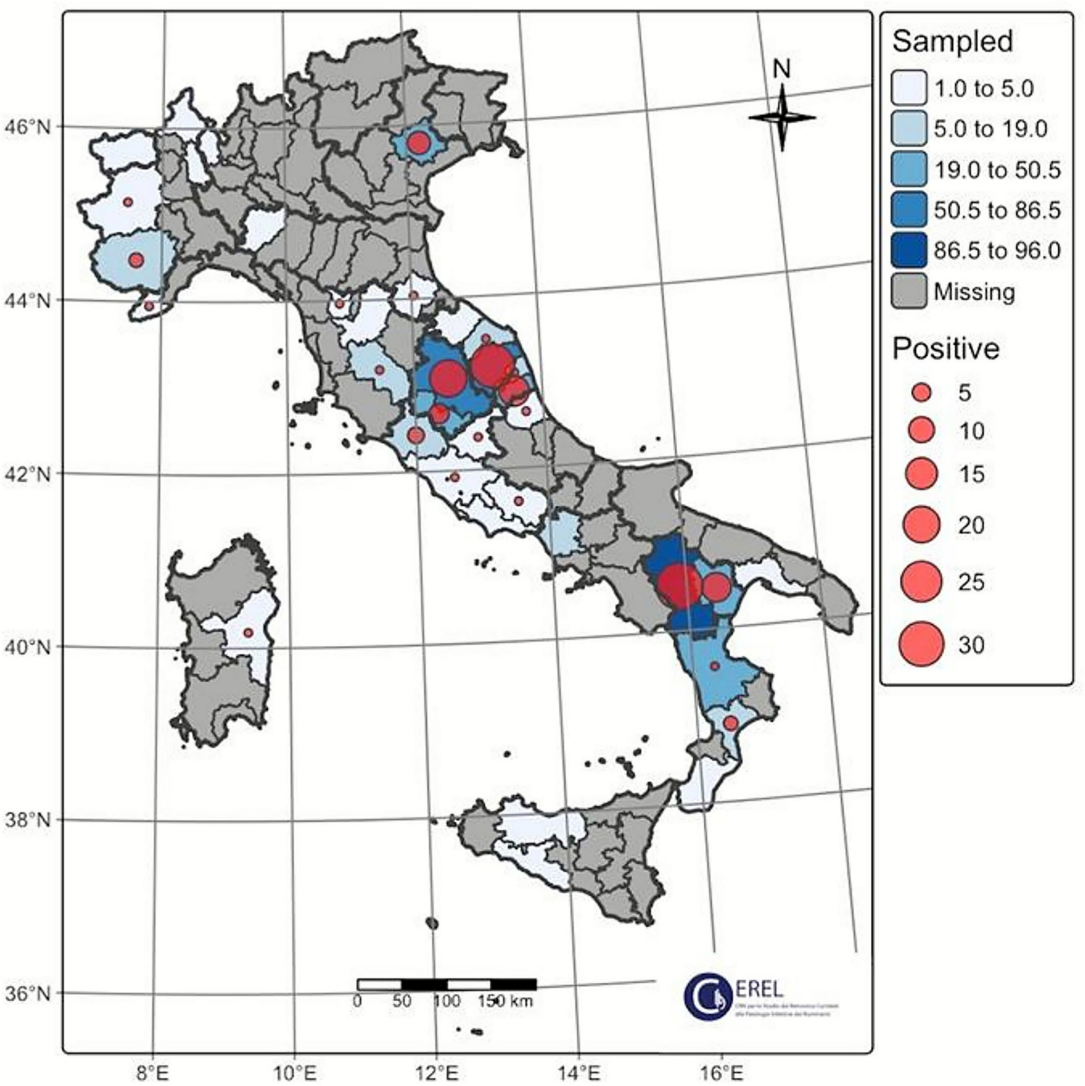
Total number of positives obtained by region and province. Fisher’s method was used to plot data.

**Figure 4:**
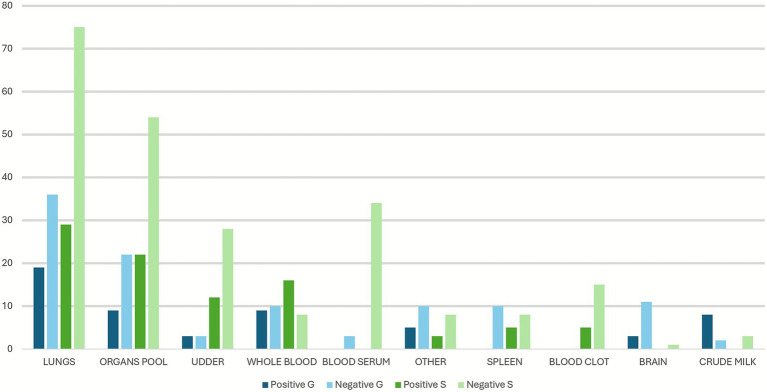
Types of analyzed matrices per species and results. G: goats, S: sheep. The number of tested samples is reported on the *Y*-axis.

During 5 years of passive surveillance (1 October 2019 to 31 October 2024) 271 ovi-caprine farms were tested for SRLV, for a total of 474 sampling events. One hundred forty eight out of 489 samples (30%) collected from 64/271 farms (23.52%) resulted positive. In detail, 56/326 (17.17%) samples from goats and 92/163 (56.44%) from sheep resulted positive. Ovi-caprine farms were distributed in 16 out of 20 Italian regions, across 38 provinces. The highest number of samples were received in three distinct years: 2019, 2022 and 2024 (Figure 1). Notably, the highest percentage of positive samples per year was observed in 2022, reaching 39.2%. From 2019 to 2024, the number of tested farms per region varied with three regions located in North, Centre and South of Italy namely: Piedmont, Umbria and Basilicata, submitting samples every year and some regions occasionally. The highest number of ovi-caprine farms were tested in 2019 (*n* = 89, 32.84%), followed by 2020 (*n* = 51, 18.81%), and 2024 (*n* = 47, 17.34%) (Figure 2). The majority (71.21%) of tested farms were located in three regions (Basilicata, Umbria and Marche) and five provinces (Figure 3). Twenty-seven farms were tested repeatedly, of which 21 farms were tested twice, five farms three times and one four times. The Basilicata region in South Italy submitted the highest number of samples, primarily in the last 3 months of 2019, followed by Marche and Umbria regions in central Italy (Figure 3). Lungs constituted the most tested sample (*n* = 111; 30.58%), followed by pools of organs (*n* = 81; 22.31%), mammary gland (*n* = 43; 11.85%) and blood (*n* = 34; 9.37%) (Figure 4).

### 3.2 The Italian SRLVs display a high *gag-pol* genetic diversity

**Figure 5:**
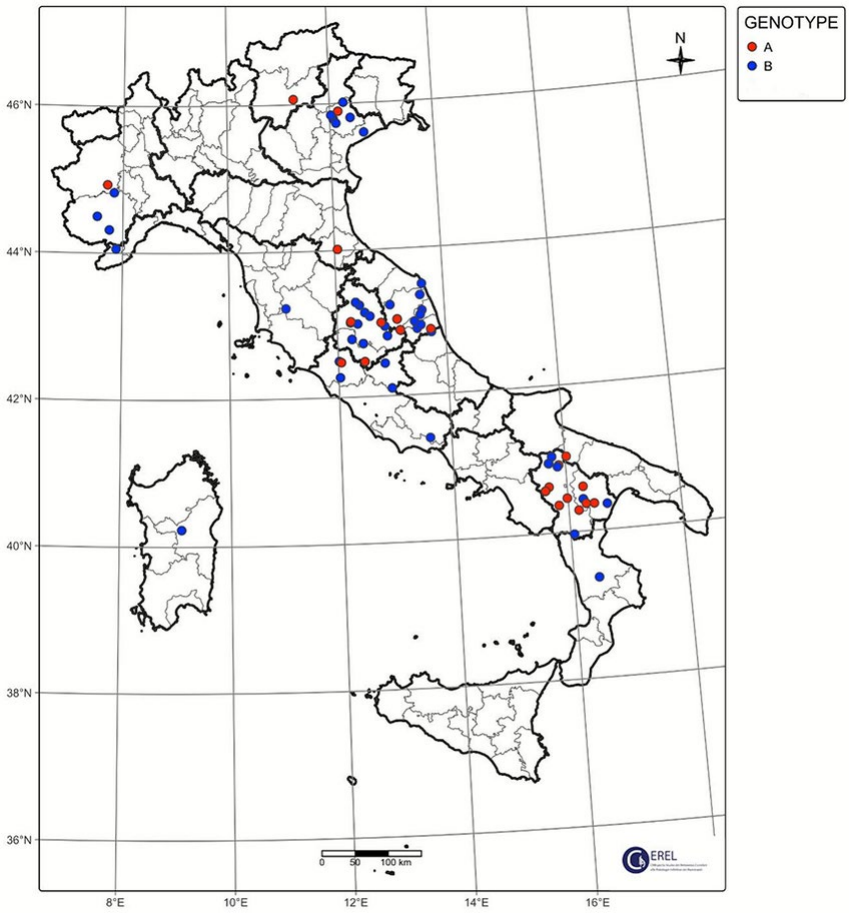
Genotyped samples between 2019–2024 and their geographic distribution.

**Figure 6:**
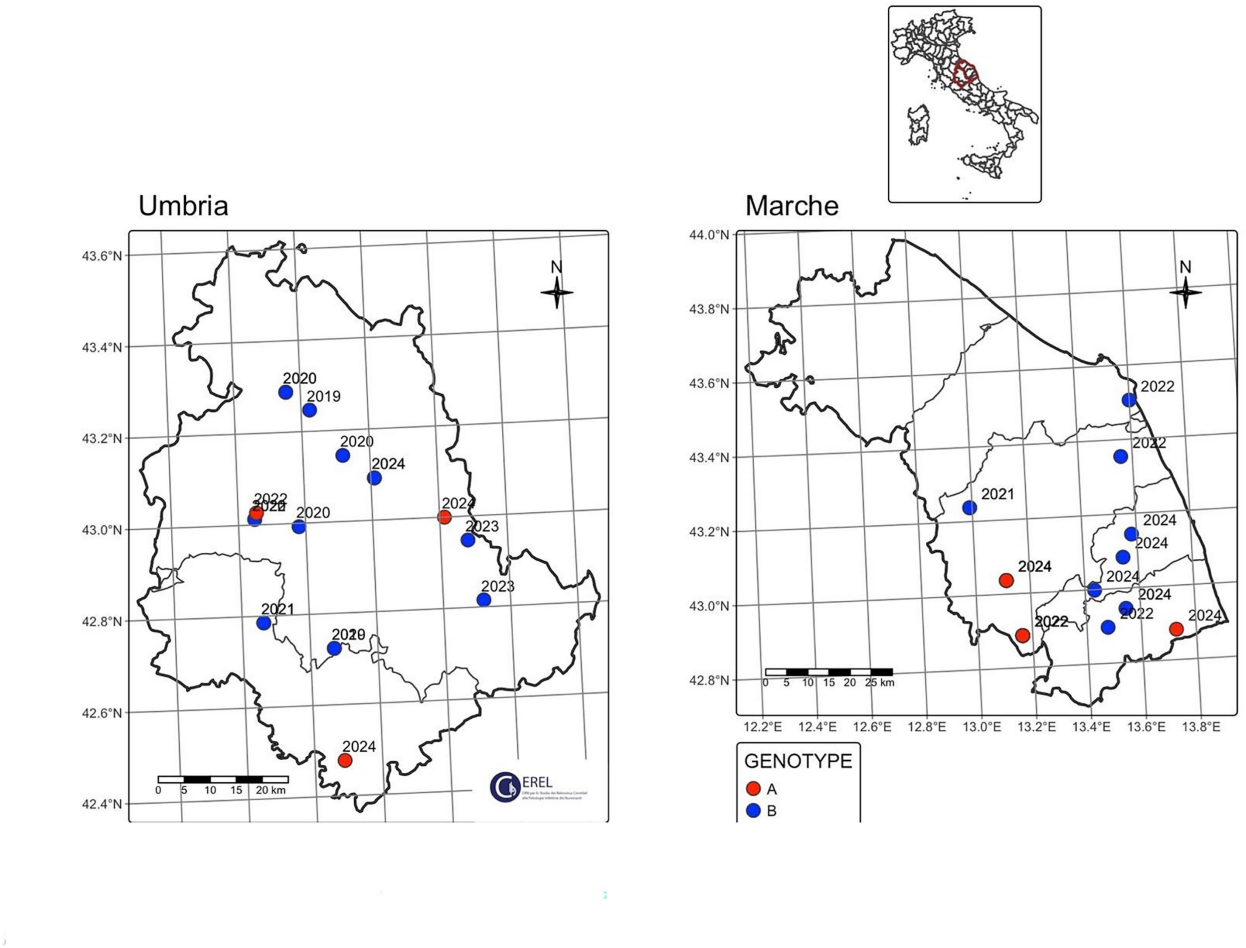
Focus on Umbria and Marche regions, sequenced samples divided per year.

**Figure 7:**
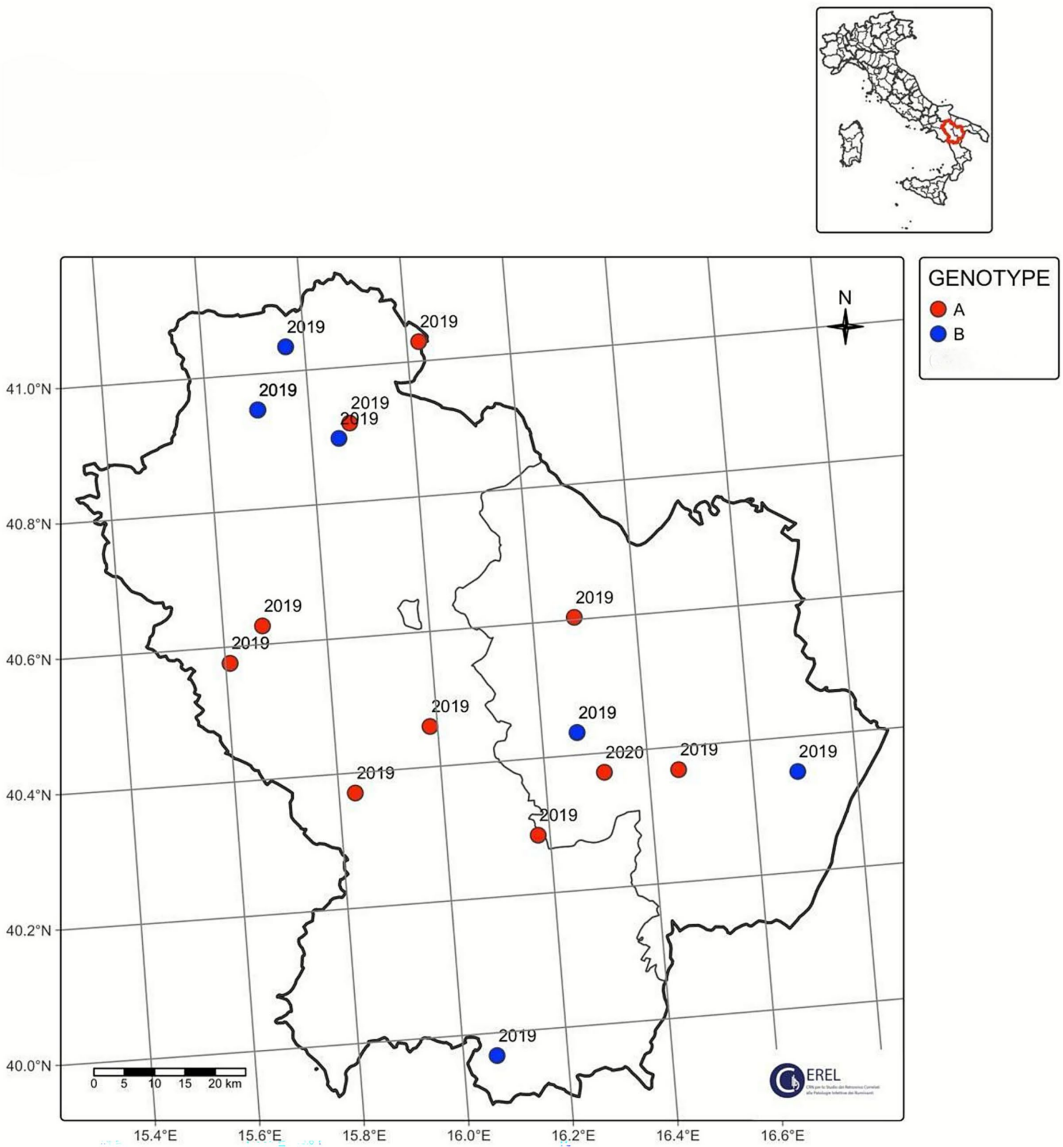
Focus on Basilicata region, sequenced samples divided per year.

Ninety out of 148 positive samples (60.8%) were successfully sequenced, belonging to 64 different farms located in 13 Italian regions, namely Abruzzo, Basilicata, Calabria, Emilia-Romagna, Lazio, Liguria, Marche, Piedmont, Sardinia, Tuscany, Trentino Alto-Adige, Umbria and Veneto (Figure 3). The largest number of successfully sequenced samples originated from four regions in south, central and North of Italy: Basilicata (*n* = 25), Marche (*n* = 23), Umbria (*n* = 18) and Veneto (*n* = 7) (Table 1 and Figure 5). The majority of sequenced SRLV were detected in 2019 (*n* = 26, 28.88%) of which 24 from Basilicata, followed by 2022 (*n* = 18, 20%) with the majority collected in Central Italy, specifically from the Marche region (Table 2 and Figures 6, 7). Considering the whole study period 2019–2024, for the first time a positive sample was sequenced from the Abruzzo region in central Italy.

**Table 1:** Distribution of genotypes identified in this study divided per region.

Regions	Genotypes	
	A	B
Abruzzo	0	1
Basilicata	11	14
Calabria	0	1
Emilia-Romagna	1	0
Lazio	1	5
Liguria	0	1
Marche	11	12
Piedmont	1	3
Sardinia	0	1
Tuscany	0	1
Trentino	1	0
Umbria	3	15
Veneto	1	6
Total	30	60

**Table 2:** Number of SRLV genotyped per year.

Genotype	2019	2020	2021	2022	2023	2024	Total per genotype
Total genotype A	10	2	1	7	1	9	30
Total genotype B	16	12	9	11	5	7	60
Total per year	26	14	10	18	6	16	90

**Figure 8:**
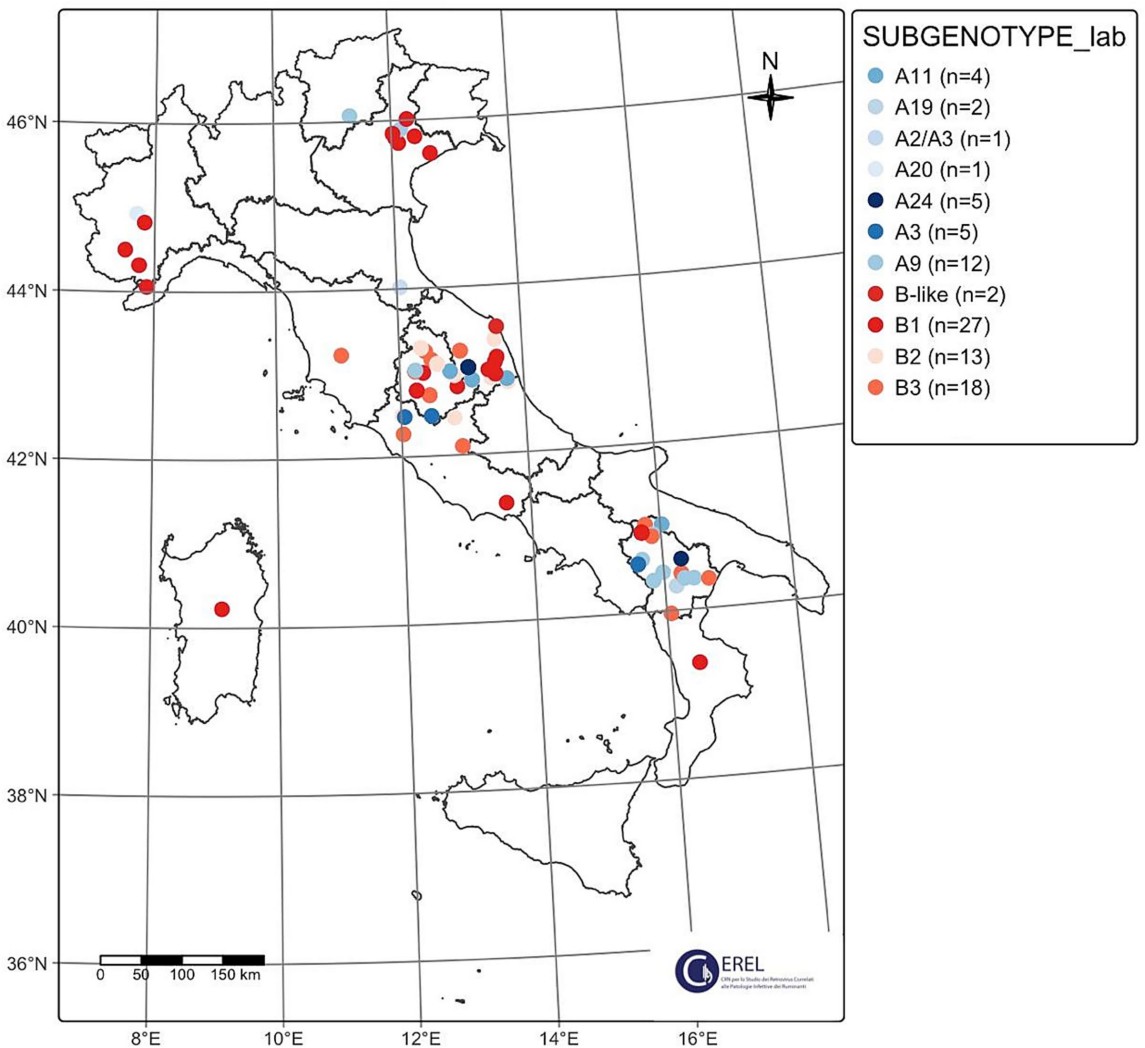
Geographical distribution of SRLV sub-genotypes over the Italian territory from 2019 to 2024.

The majority of the characterized SRLV belonged to the B genotype (*n* = 60, 66.66%) and the remaining to genotype A (*n* = 30, 33.33%), with no detection of any other SRLVs genotypes. SRLVs samples belonging to the A and B genotypes were collected across Italy in eight and 11 Italian regions, respectively (Table 1). Among the most surveyed regions, Umbria, Marche and Basilicata, only Umbria presented the majority of characterized samples belonging to genotype B (Table 1). All the B sub-genotypes (B1–3) and 7 A sub-genotypes (Supplementary Table 1 and Figure 8) were detected during the study period according to the clustering used by previous studies, while two SRLV strains remain unassigned. In detail, sequenced samples belonged to sub-genotypes B1 (*n* = 26), B2 (*n* = 13), B3 (*n* = 19) and were detected in 10 out of 13 regions. Samples belonging to genotype B1 were collected in nine different regions between 2019 and 2024 and appeared to circulate widely across Italy, including Sardinia Island. Among the B sub-genotypes, the B1 was the only one detected also in northern regions (Liguria, Piedmont and Veneto). Samples belonging to the B2 sub-genotype were detected only in central Italy (Abruzzo, Lazio, Marche and Umbria), between 2020 and 2024 (Supplementary Table 1 and Figure 8). Finally, the B3 sub-genotype was detected in five regions of central and southern Italy only (Tuscany, Marche, Basilicata, Lazio and Umbria) (Supplementary Table 1 and Figure 8). The A genotype SRLVs were classified as A3 (*n* = 5), A2/3 (*n* = 1), A9 (*n* = 12), A11 (*n* = 3), A19 (*n* = 2), A20 (*n* = 1), and A24 (*n* = 4), and two unassigned, according to the currently existing SRLVs genetic classification. The A9 SRLVs were primarily collected in Central (Marche region) and South (Basilicata region) Italy.

Based on the phylogenetic tree, some of the sequences obtained in the present study cluster within groups that, according to the analyses presented in this paper, do not appear to be monophyletic or show low bootstrap support. This is the case for sub-genotype A24, whose samples cluster into four individually well-supported groups, while the current A24 sub-genotype does not seem to be supported by the inferred topology. Sub-genotype A3 exhibits low bootstrap support (<70) and appears to include the whole A2 sub-genotype. On the other hand, sub-genotypes A9 and A19 form a moderately supported monophyletic group. Interestingly, based on monophyly and bootstrap support, sub-genotype A9 can be further divided into two additional monophyletic and well-supported clades.

**Figure 9:**
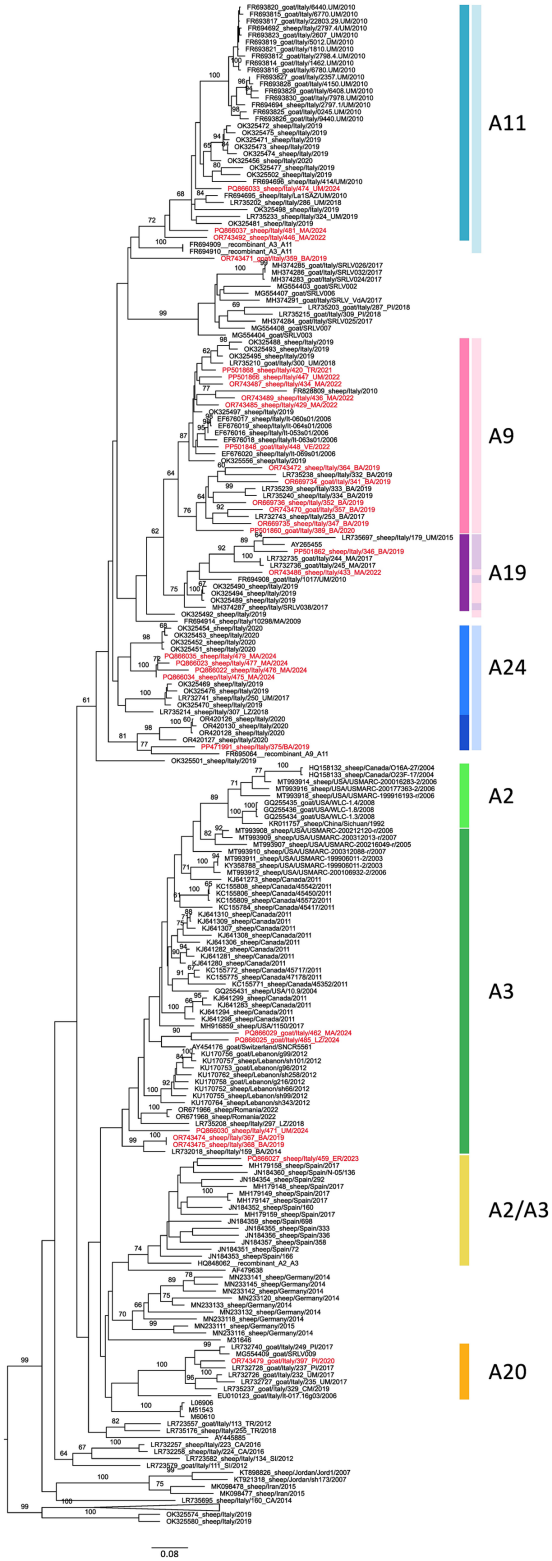
Phylogenetic tree associated with genotype A, based on the sequencing of the 800 bp *gag-pol* region. Sequences obtained in this work are highlighted in red. Colored bars indicate the sub-genotypes assigned to the sequences included in this study. Lighter shades correspond to previously published classifications.

The topology of the phylogenetic tree of SRLV belonging to genotype A showed two major groups within genotype A (Figure 9). A first major group consisted of SRLVs sequences that fell within the historically identified A11, A9, A19 and A24 sub-genotypes, including the newly sequenced samples PP471991_sheep/Italy/375/BA/2019 and OR743471_goat/Italy/359_BA/2019, temporarily not assigned to any described sub-genotype (Figure 9). A second major group appeared composed of seven sequences assigned to A3, A2/3 and A20 sub-genotypes. In detail, the SRLVs belonging to A11 sub-genotype form a well-supported group, including Italian SRLVs previously characterized in 2010 and 2019. This strain appeared distantly related to the A11 and more closely related to SRLV previously identified as recombinant strains (Figure 9). Interestingly, the majority of the A11 Italian SRLV originated in central Italy: Marche region (Figure 9). In addition, sample PQ866037_sheep/Italy/481_MA/2024 may represent the outcome of a recombination event involving strains from sub-genotypes A19 and A11.

Twelve SRLVs sequences generated in this study clustered with sequences classified as A9. This cluster appeared to be divided into two well-supported subgroups: one including SRLVs identified in 2022 from central and northern Italy together with Italian SRLVs identified since 2006 and the other comprising SRLVs originating exclusively from the Basilicata region and collected mainly in 2019 (Figure 9). Both sub-groups showed high genetic diversity, with average pairwise distances of 6.9 and 11.8% for the former and the latter, respectively.

Sample PP471991_sheep/Italy/375/BA/2019 clustered with sequences previously assigned to sub-genotype A24, collected in Basilicata in 2020. Nevertheless, considering both the inferred phylogenetic topology and the average pairwise distance (13.8%) from the other A24 sequences, this entire subgroup may warrant designation as a new sub-genotype. Based on the topology of the phylogenetic tree, the second major group is composed of SRLV described as A3, A2/A3 and A20. One small group identified as belonging to A3 sub-genotype includes SRLVs sequenced in this study, in southern Italy (Basilicata) in 2019, together with an SRLV collected in central Italy in 2014 (Figure 9). SRLVs from Spain and one Italian SRLV from northern Italy (PQ866027_sheep/Italy/459_ER/2023) (Figure 9) mainly composed the A2/A3 sub-group. The third sub-group is composed of SRLVs identified as A20, primarily from Italy, identified between 2009 and 2020 (Figure 9).

**Figure 10:**
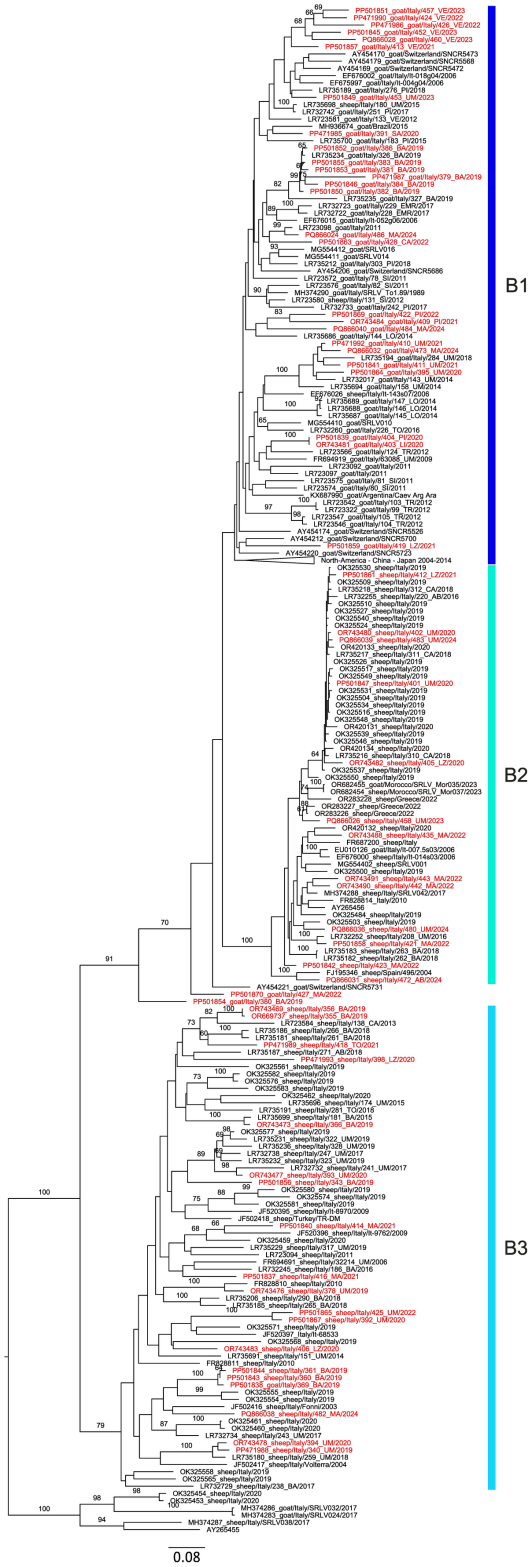
Phylogenetic tree associated with genotype B, based on the sequencing of the 800 bp *gag-pol* region. Sequences obtained in this work are highlighted in red. Colored bars indicate the sub-genotypes assigned to the sequences included in this study.

Based on phylogenetic analysis, genotype B displays three well-defined sub-genotypes namely: B1, B2 and B3 (Figure 10), with B1 and B2 clustering together. The average pairwise distance (APD) between sub-genotypes B1 and B2 was 14%, whereas both B1 and B2 showed an APD of 20% when compared with sub-genotype B3.

Interestingly, two SRLVs sequenced in the present study (PP501854_goat/Italy/380_BA/2019 and PP501870_goat/Italy/427_MA/2022) are closely related to B1 and B2 sub-genotypes, but they fall outside the main node containing both B1 and B2 (Figure 10). Looking at the pairwise distances, the two samples displayed an APD of 17 and 13.5% with the B1 sub-genotype and of 18.2 and 15% with the B2 sub-genotype, respectively, thus resulting more similar to the B1 samples.

Within the B1 sub-genotype, subgroups formed by samples from the same geographic origin were identified. This was the case of five samples collected between 2021 and 2023 in Veneto and those from Basilicata in 2019, to which sample LR735234_goat/Italy/326_BA/2019 also from the same region can be included. In both cases, the subgroups were supported by bootstrap values (Figure 10). Other small, supported subgroups within B1 included SRLVs from central Italy (Umbria and Marche) collected between 2014 and 2024 with two SRLVs identified in the same farm in different months (PP471992_goat/Italy/410_UM/2021 and PP501841_goat/Italy/411_UM/2021) and the two 2020 SRLV from Liguria and Piedmont (OR743481_goat/Italy/403_LI/2020 and PP501839_goat/Italy/404_PI/2020) (Figure 10). The genetic variability of the B3 sub-genotype appeared higher compared to sub-genotypes B1 and B2, as B3 displays a within-group APD of 14.2% while B2 and B1, 5.7 and 11.7%, respectively. Moreover, SRLVs collected in the same region clustered together only sporadically (Figure 10). Interestingly, in the B3 sub-genotype fell one highly supported group composed only by three SRLVs strains, all of which identified in the same farm in three consecutive years (2018–2020) (PP471988_sheep/Italy/340_UM/2019, OR743478_sheep/Italy/394_UM/2020, and reference sequence LR735180_sheep/Italy/259_UM/2018) (Figure 10). The same was observed for the PP501865_sheep/Italy/425_UM/2022 and PP501867_sheep//Italy/392_UM/2020, that grouped together and were identified in the same farm in central Italy 2 years apart (Figure 10).

**Figure 11:**
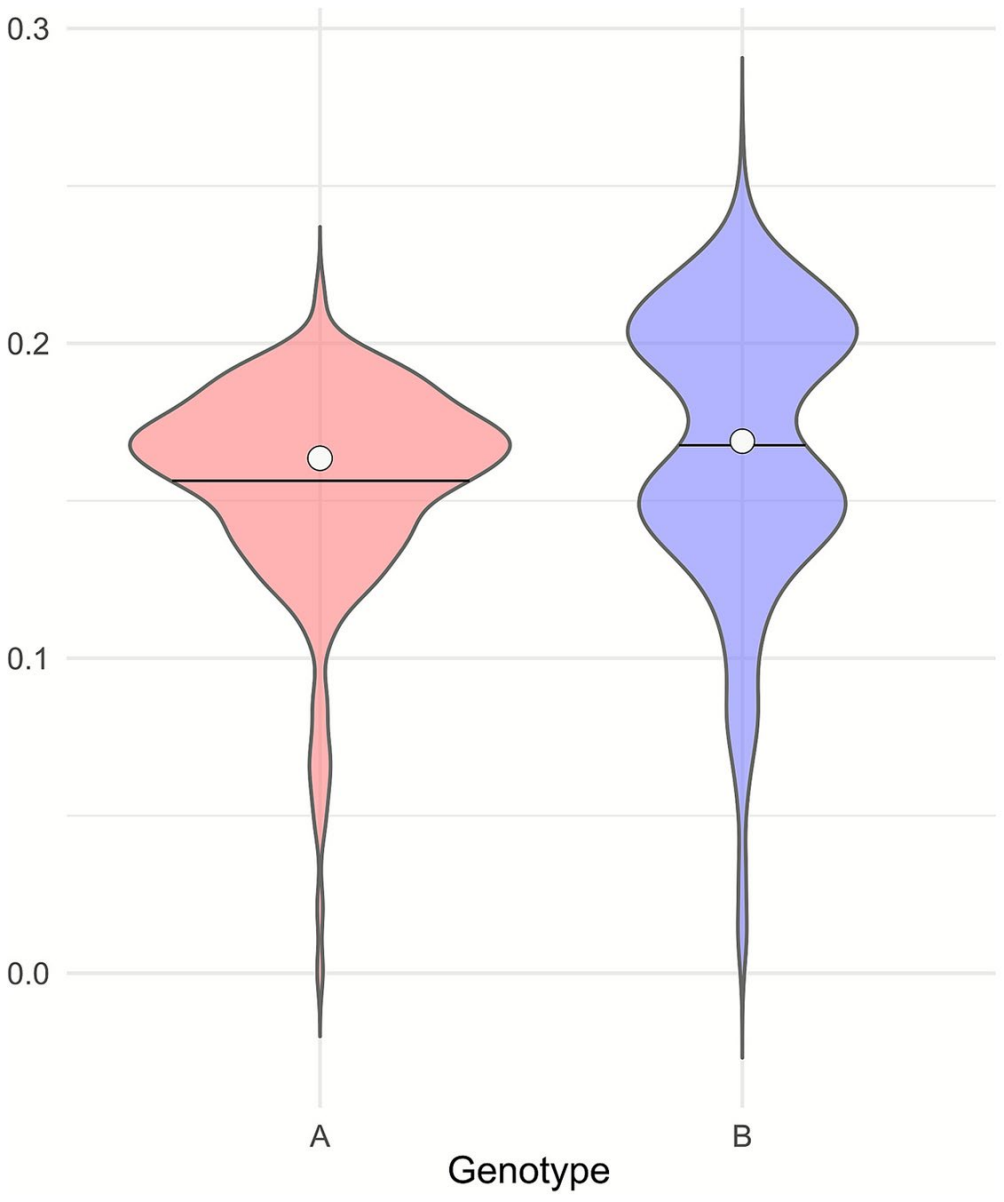
Violin plots representing the p-distances distribution of genotypes A and B.

Based on the pairwise p-distances calculated from the sequences obtained in this study, we observed that the two genotypes, A and B, show comparable levels of genetic diversity (Figure 11). Sequences obtained in this study belonging to genotype A displayed a mean p-distance of 15.6 ± 3.2%, while those of genotype B showed a mean p-distance of 16.7 ± 4.4%. The maximum p-distances observed were 21.6% for genotype A and 26.4% for genotype B.

## 4 Discussion

In the absence of mandatory control and eradication programs in several countries, including Italy, the genetic characterization of SRLVs largely relies on data from passive surveillance (Kalogianni et al., 2020; Carrozza et al., 2023; Colitti et al., 2019). The present study allowed the characterization of recent Italian SRLVs by Sanger sequencing. During 5 years of passive surveillance (end of 2019–2024), a total of 90 SRLVs were genetically characterized across Italy, thus enhancing and updating the current knowledge on the genetic information available on circulating SRLVs.

As the study relied on passive surveillance data gathered over 5 years, its representativeness at the national level may be limited. Sampling events were not uniformly distributed across the country, varying in number, frequency, and geographic location. Some regions, particularly in central and southern Italy, contributed disproportionately with more samples. This uneven distribution led to a higher detection rate of SRLV-positive cases in those areas, which in turn allowed for a more detailed characterization of the regional genetic diversity of SRLVs. This was especially evident in the Basilicata region (southern Italy) and in the Umbria and Marche regions (central Italy). Moreover, a higher number of positive cases were identified in sheep compared to goats, which may be explained by the different species susceptibility reported in previous studies (Ramírez et al., 2013; De la Luz-Armendáriz et al., 2025; Heaton et al., 2012; Sider et al., 2013). Nevertheless, despite the non-uniform sampling typical of passive surveillance systems, the sequenced SRLVs strains still included representatives from northern, central, and southern regions of Italy, providing an initial yet informative picture of the genetic diversity of SRLVs across the country.

This study confirmed the circulation of A and B genotypes in Italy, with no detection of other genotypes, in line with previous studies (Bazzucchi et al., 2021; Arcangeli et al., 2022). Sequenced SRLVs presented a high genetic diversity, as suggested by the topology of the phylogenetic trees and confirmed by the calculated p-distances, confirming previous evidence (Bazzucchi et al., 2021; Arcangeli et al., 2022; Olech et al., 2022b). Comparing the two genotypes and looking at the sequences obtained, A and B exhibited comparable mean p-distances, suggesting similar levels of overall genetic diversity during the study period. Nevertheless, genotype A sequences are distributed across a greater number of sub-genotypes, reflecting the fact that genotype A has historically undergone a more extensive subdivision than genotype B. The phylogenetic analysis conducted on Italian SRLVs clearly suggested the circulation of two main genetic groups within the A genotype.

Furthermore, the phylogenetic analysis demonstrated that SRLVs sequenced previously assigned to the same A sub-genotypes grouped together with low or insufficient bootstrap values and therefore cannot be robustly defined as distinct sub-genotypes. This behavior observed for SRLVs, included in our analysis, and described by other authors, as belonging to A2/3, A3 and A24 sub-genotypes. Interestingly the A24 sub-genotype was firstly proposed by Bazzucchi et al. (2021) in Italy (Ostuni et al., 2024), as a potential new sub-genotype and subsequently described in other European countries such as Poland (Olech and Kuźmak, 2021; Olech et al., 2022a; Olech and Kuzmak, 2024) and Greece (Kalogianni et al., 2024). Furthermore, phylogenetic analysis unambiguously revealed cases of misclassification. This is also the case for some samples (reference sequences FR694908_goat/Italy/1017/UM/2010, OK325489_sheep/Italy/2019, OK325490_sheep/Italy/2019, OK325494_sheep/Italy/2019) previously identified as A9, which clearly belong to the A19 sub-genotype. This suggests that the current sub-genotype classification does not depict the existing genetic diversity of Italian SRLVs and therefore posing questions on its suitability. Some of the A strains characterized appeared to cluster together based on the region of origin as the A9 sub-group formed by SRLVs circulating in the Basilicata region. Although further observations are necessary, this evidence may indicate a region-based diversity potentially influenced by yet unexplored local factors.

The observed discrepancies could be due to different alignment parameters, choices made during the manual refinement or inclusion of new samples. In addition, the use of a relatively short genomic region encompassing a hypervariable segment may contribute to a weakened phylogenetic signal. This limitation could be partially overcome by analyzing longer sequence fragments. Further sequencing and additional analyses may help elucidate the real relationship between circulating SRLVs.

The B genotype was detected every year and in many surveyed regions, suggesting its wider distribution compared to genotype A in Italy during the study period. This evidence may reflect the ancient divergence between A and B genotypes, which was associated with distinct geographic origins: genotype A likely originated from Central Asia whereas genotype B appears to have emerged in continental Europe (Carrozza et al., 2023). Interestingly, the study revealed that sub-genotype B1 was widespread across Italy, while B2 appeared to be mostly restricted to central regions. Conversely, B3 appeared to be limited to southern Italy. Nevertheless, B2 strains were also detected in southern Italy in 2018 (Bazzucchi et al., 2021) and B3 was first reported in 2003 (Bertolotti et al., 2011). The wider distribution of the B1 sub-genotype was confirmed in other European countries as well (Olech, 2023). Whether such distribution reflects a region-based genetic distribution of SRLVs in Italy remains speculative and requires further investigations. This study demonstrated, for the first time, the existence of SRLVs with genetic characteristics that do not correspond to any of the identified B sub-genotypes and herein referred to as B-like SRLVs. The B1 SRLVs form small genetic clusters that reflect the region and/or farm of origin. A similar situation was observed for one B3 cluster supported by high bootstrap values, which was composed of SRLVs detected in the same farm over a three-year period. These findings suggest that continuous monitoring, together with genetic characterization of positive SRLVs cases, could provide insights into the distribution and genetic characteristics of the B-like SRLVs, as well as the mechanisms of spread and maintenance of the infection at regional/farm level.

Regarding the sub-genotype presence and distribution, our study only partially confirmed previous studies (Olech, 2023). All sequenced SRLVs, with the exception of the B-like ones, were classified in one of the existing sub-genotypes previously described in Italy (Bazzucchi et al., 2021; Arcangeli et al., 2022). Up until 2019, Italy had already identified sub-genotypes B1, B2, and B3 (Giammarioli et al., 2011). It is possible to say the same for sub-genotypes A9, A19, A11, A20, A24 (Bazzucchi et al., 2021; Grego et al., 2007; Colitti et al., 2019). Therefore, the samples classified in this study are in genetic continuity with those previously described, highlighting how these genotypes continue to circulate in Italy. With regard to classification into sub-genotypes, we have adopted the nomenclature proposed in the literature (Shah et al., 2004a). However, it should be noted that the topology observed for some sub-genotypes did not agree with that described in the literature (Bazzucchi et al., 2021; Arcangeli et al., 2022; Olech et al., 2022b). The discrepancies observed in sub-genotype classification underscore the need for continued genetic characterization of circulating SRLVs, through full gene and possibly whole genome sequencing, in order to enhance the accuracy and robustness of the proposed genetic classification (Shah et al., 2004a; L’Homme et al., 2011). Such approaches should be implemented further for SRLV challenging their significant genetic diversity and low copy counts. Based on phylogenetic analysis of SRLVs complete genomes it was possible to deduce that their wide distribution was a result of both ancient and more recent recombination events (Carrozza et al., 2023). The high genetic variability combined with the limited number of complete genomic sequences (less than 100 globally) poses significant challenges to the development of effective molecular detection and characterization tests. Despite the economic and cultural significance of sheep and goat husbandry in Italy, this constraint makes it difficult to fully understand infection dynamics and epidemiological trends, which leads to the ongoing neglect status of Visna-Maedi/CAEV among the animal diseases (Gobbi et al., 2024).

## 5 Conclusion

In conclusion, data generated indicated that appreciable and stable genetic differences exist between genotypes A and B, and that the classification into sub-genotypes, particularly within genotype A, may lead to an excessive diversification not supported by genetic evidence. Therefore, our study shed light for the first time on the real genetic diversity between A and B genotypes and on the opportunity to rethink the available genetic classification into sub-genotypes based on the *gag-pol* diversity. In addition, our findings support the need for ongoing surveillance of SRLVs, underscoring the importance of enhancing passive surveillance in all Italian regions to clarify the genetic ambiguities and region-based clustering observed and to gain a more comprehensive understanding of SRLVs genetic diversity.

## Data Availability

The datasets presented in this study can be found in online repositories. The names of the repository/repositories and accession number(s) can be found in the article/Supplementary material.
